# Aetiology and factors associated with bacterial diarrhoeal diseases amongst urban refugee children in Eastleigh, Kenya: A case control study

**DOI:** 10.4102/ajlm.v2i1.63

**Published:** 2013-09-03

**Authors:** Waqo G. Boru, Gideon Kikuvi, Jared Omollo, Ahmed Abade, Samuel Amwayi, William Ampofo, Elizabeth T. Luman, Joseph Oundo

**Affiliations:** 1 Field Epidemiology and Laboratory Training Programme, Kenya; 2 Institute of Tropical Medicine, Jomo Kenyatta University of Agriculture and Technology, Kenya; 3 Noguchi Memorial Institute for Medical Research, University of Ghana, Legon; 4 Center for Global Health, U.S. Centers for Disease Control and Prevention, United States; 5 Centre for Disease Control and Prevention, Kenya

## Abstract

**Introduction:**

Kenya is home to over 400 000 refugees from neighbouring countries. There is scanty information about diarrhoea amongst urban refugees in Kenya.

**Objectives:**

We investigated the enteric bacteria causing diarrhoea amongst urban refugee children and described the associated factors.

**Method:**

During the period of August–December 2010, urban refugee children between the ages of two and five who attended Eastleigh County Council Health Centre were enrolled into the study. Diarrhoeal cases were compared with age-matched children with no diarrhoea (controls). Stool specimens were collected and enteric bacteria isolated. A questionnaire was administered to identify risk factors.

**Results:**

A total of 41 cases and 41 controls were enrolled in the study. The age and country of origin were similar for cases and controls. The bacterial isolation rates amongst the cases were: non-pathogenic *Escherichia coli* 71%, *Shigella dysenteriae* 2.4%, *Shigella flexneri* 2.4%, *Salmonella paratyphi* 5%. For the controls, non-pathogenic *E. coli* 90% and enterotoxigenic *E. coli* (ETEC) 2.4% were amongst the organisms isolated. All isolates were resistant to amoxicillin; resistance to other antibiotics varied by isolate type. Factors associated independently with diarrhoea included children not washing their hands with soap (aOR 5.9, *p* < 0.05), neighbour(s) having diarrhoea (aOR 39.8, *p* < 0.05), children not exclusively breastfed for their first 6 months (aOR 7.6, *p* < 0.05) and children eating food cooked the previous day (aOR 23.8, *p* = 0.002).

**Conclusions:**

*Shigella* species, *Salmonella* species and ETEC were found to be responsible for diarrhoea amongst the urban refugee children. Measures to control and guide the use of antibiotics are critical for the prevention of antibiotic resistance. Efforts to improve personal and domestic hygiene, including educational campaigns to promote appropriate handwashing, should be encouraged.

## Introduction

Diarrhoea is the second most frequent cause of death globally amongst children aged five and below,^1^ accounting for more than one-third of the deaths in this age group – more than HIV, malaria and measles combined.^2^ More than 80% of child deaths due to diarrhoea occur in Africa and South Asia, with only fifteen countries, including Kenya, contributing to almost three-quarters of the total.^2^ In Kenya, diarrhoea is a leading cause of morbidity, accounting for 17% of childhood illnesses.^3^

Thirty per cent of the 9.2 million refugees in the world are found in sub-Saharan Africa, and of these, 80% are women and children.^4^ Kenya hosts over 400 000 refugees from its neighbouring countries.^5^ Eastleigh suburb in Nairobi is a cosmopolitan commercial centre hosting a mixed population of native Kenyans and a large proportion of urban refugees from Somalia, Eritrea and Ethiopia. There is also a constant bidirectional movement of people between this suburb and the two refugee camps, Kakuma and Dadaab, located in the northern part of Kenya.

Previous studies on diarrhoea amongst refugee populations in Kenya were focused solely on the situation in the camps. There is, however, an increasingly large number of refugees dispersed in the urban areas. There is also limited information on the causes and associated risk factors of diarrhoea in the urban refugee children. For this study, a health facility-based, matched case–control study was conducted to determine bacterial aetiology and associated risk factors for diarrhoea amongst young urban refugee children in Eastleigh, Nairobi.

## Research method and design

### Study site

Eastleigh is part of the Pumwani division of Nairobi with an approximate area of 7.4 km^2^ and a population of approximately 200 000 people.^3^ This study was conducted at Eastleigh City Council Health Centre, where refugees are able to access free medical services through the support of the German Technical Cooperation Agency.

### Study population

Children who were registered with the United Nations High Commission for Refugees, were between two and five years of age and resided in Eastleigh were enrolled in the study.

### Definition of cases and controls

Children seen in Eastleigh City Council Health Centre with diarrhoea from August–December 2010 who had not started any antibiotic treatment were recruited as cases. Controls were hospital-based and were defined as children who had sought medical attention for conditions other than diarrhoea, had no history of diarrhoea in the previous four weeks (based on the report of their caregiver) and were not on antibiotics. These children were recruited as controls upon informed and voluntary consent by their caregiver. The cases were then categorised as acute (defined as an episode lasting less than two weeks), persistent (diarrhoea lasting two weeks or longer) and bloody (defined as gross appearance of blood in the stool,^6^ as reported by the parent or guardian or noted by the study personnel, regardless of duration).

### Sampling

The sample size was determined using an assumed prevalence of 32% for bacterial aetiologies in children below the age of five years as described in previous studies done in sub-Saharan Africa.^7^ Fleiss’s formula^8^ was used to generate the sample size, at a confidence interval of 95%. This yielded a sample size of 82 participants (41 cases and 41 controls). Systematically, every second child meeting the case definition was enrolled in the study. Controls were selected as the next age-matched child (±5 months from the age of a case) meeting the inclusion criteria who attended the health facility during the study period.

### Data and stool sample collection

We interviewed parents and guardians of the eligible participants using a standard questionnaire that addressed demographic and epidemiological data, duration of exclusive breastfeeding, hygiene practices, source and storage of drinking water and disposal of faeces. However, clinical information relevant to diarrhoea was obtained exclusively from the cases.

Stool specimens were collected using sterile plastic containers and transported in Cary-Blair Transport Media to the laboratory at the Centre for Microbiology Research (CMR) at the Kenya Medical Research Institute (KEMRI) for bacterial culture and antimicrobial susceptibility testing. Specimens were investigated for the isolation of enteric bacterial pathogens using conventional methods.^9^ All isolates identified as *Escherichia coli* were characterised further by means of multiplex polymerase chain reaction (mPCR) targeting specific genes with the primers outlined in Table 1. The PCR allowed for characterisation of the *E. coli* isolates as enterotoxigenic (ETEC), enteropathogenic (EPEC), enterohaemorrhagic (EHEC), enteroinvasive (EIEC) or enteroaggregative (EAEC).^10^ The inocula for susceptibility testing were compared against the McFarland 0.5 turbidity standard with the *E. coli* ATCC 25922 strain being used as the test standard. Antimicrobial susceptibility testing was done on Mueller-Hinton agar using the Etest^®^ Minimum Inhibitory Concentration (MIC) method. The interpretation of results was according to Clinical Laboratory Standard Institute (CLSI) guidelines.^11^ The following antimicrobials were tested: amoxicillin, ampicillin, gentamycin, tetracycline, chloramphenicol, kanamycin, ciprofloxacin, ofloxacin, nalidixic acid, erythromycin, ceftriaxone and trimethroprim-sulphamethaxazole.

**TABLE 1:** Primers sequence and target genes in the multiplex polymerase chain reaction (PCR) in the detection of diarrhoeagenic *Escherichia coli*.

Primer	Target gene	Primer sequence	Amplicon Size (bp)
LT	*eltB*	5’-TCTCTATGTGCATACGGAGC-3’ 5’-CCATACTGATTGCCGCAAT-3’	322
ST	*estA*	5’-GCTAACCAGTAGAGGTCTTCAAAA-3’ 5’-CCCGGTACAGAGCAGGATTACAACA-3’	147
VTI	*vt1*	5’-GAAGAGTCCGTGGGATTACG-3’ 5’-AGCGATGCTATTAATAA-3’	130
VT2	*vt2*	5’-ACCGTTTTTCAGATTTGACACATA-3’ 5’-TACACAGGAGCAGTTTCAGACAGT-3’	298
Eae	*eaeA*	5’-CACACGAATAAACTGACTAAAATG-3’ 5’-AAAAACGCTGACCCGCACCTAAAT-3’	376
SHIG	*Ial*	5’-TGGTAGGTATGGTGAGG-3’ 5’-CCAGGCCAACAATTATTTCC-3’	320
bfpA	*bfpA*	5’-TTCTTGGTGCTTGCGTGTCTTTT-3’ 5’-TTTTGTTTGTTGTATCTTTGTAA-3’	367
EA	pCVD	5’-CTGGCGAAAGACTGTGTATCAT-3’ 5’-CAATGTATAGAAATCCGCTGTT-3’	630

*Source:* Nguyen TV, Phung LV, Chinh LH, et al.^26^ LT, Labile Toxin; ST, Stable Toxin; VTI, Verocytotoxin 1; VT2, Verocytotoxin 2; Eae, Attaching and Effacing e.coli; SHIG, Shiga toxin; bfpA, Bundle forming Pilli A; EA, E.coli thioredoxin A; bp, base pairs.

### Statistical analysis

Data analysis was performed using Epi Info version 3.4.3 (CDC, Atlanta, USA) software. Bacterial isolation rates were calculated for both cases and controls and proportions were compared using the Chi-square test corrected for matched case control. All variables with *p*≤0.1 in bivariate analysis were included in the initial multivariable conditional logistic regression model. Standard backward elimination was conducted in order to obtain a final model of factors that were associated independently with diarrhoea at a *p* value of < 0.05.

## Ethical considerations

The study was approved by the Jomo Kenyatta University of Agriculture and Technology and the National Ethical Review Board at KEMRI. Written informed consent was obtained from the parents or guardians of the children enrolled in the study.

## Results

### Demographic characteristics of study participants

The 41 cases and 41 controls were similar with respect to their mean age, 38 versus 37 months respectively. The age range was 24–59 and 24–55 months for cases and controls respectively. Most of the study participants were from Somalia (73% of cases and 51% of controls), with the remainder from Ethiopia (27% of cases and 34% of controls) and Eritrea (0 cases and 15% of controls). Seven cases (17%) had received some form of treatment prior to arrival at Eastleigh City Council Health Centre. Most of the cases (78%) were diagnosed with gastroenteritis, whilst the remaining were diagnosed with dysentery (2%), malaria (5%) or other conditions (6%) (Table 2). Ninety-three per cent of the cases had acute diarrhoea. The majority of the cases were prescribed antibiotics (76%) and/or flagyl (78%) (Table 2).

**TABLE 2:** Clinical information of urban refugee children with diarrhoea between the ages of two and five seen at Eastleigh City Council Health Centre, Nairobi, August–December, 2010.

Clinical information of the cases (*n* = 41)	Frequency	95% C.I	
		LL	UL
**Treatment before attending health centre**		
Yes	7 (17%)	7.2	32.1
No	34 (83%)	67.9	92.8
**Dehydration (*n* = 40)**		
Yes	3 (7%)	1.6	20.4
No	37 (93%)	79.6	98.4
**Clinical diagnosis**		
Gastroenteritis	32 (78%)	62.4	89.4
Dysentery	1 (2%)	0.1	129
Malaria	2 (5%)	0.6	16.5
Others*	6 (15%)	5.6	29.2
**Treatment at the health facility**		
Antibiotics	31 (76%)	59.7	87.6
Oral rehydration salts	12 (30%)	16.1	45.5
Antimalarials	9 (22%)	10.6	37.6
Flagyl (antiamoebicide)	32 (78%)	62.4	89.4
**Type of diarrhoea**		
Acute diarrhoea	38 (93%)	81.2	96.4
Bloody diarrhoea	1 (2%)	0.3	13.6
Persistent diarrhoea	2 (5%)	1.4	18.1

*Source*: Authors’ own construction

* Others in clinical diagnosis – Helminthiasis, amoebiasis and respiratory infections; LL, Lower Limit; UL, Upper Limit; CI, Confidence Interval.

### Bacterial isolates

Most of the isolates obtained (29 [71%] cases and 37 [90%] controls) were non-pathogenic *E. coli*. The pathogenic isolates were isolated from seven cases and one control. Enterotoxigenic *E. coli* was the highest-ranking isolate, with three from the cases and one from a control. Only two *Shigella* spp. and *Salmonella* spp. isolates were obtained from the cases.

### Antimicrobial susceptibility pattern

**FIGURE 1:**
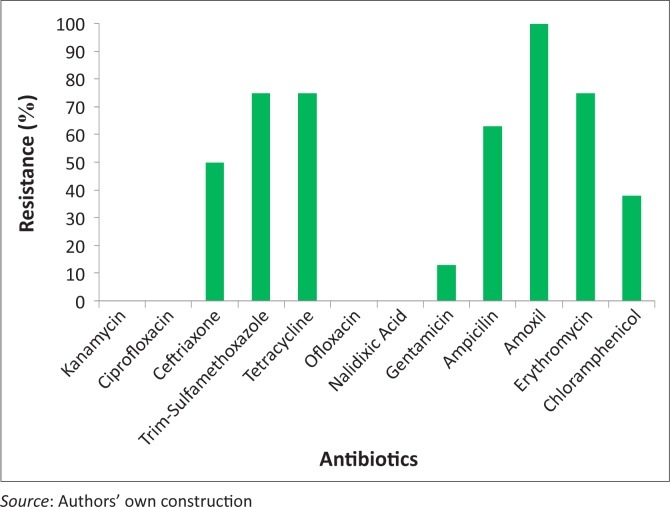
Antimicrobial resistance patterns of pathogenic isolates among urban refugee children in Eastleigh in Nairobi, August–December, 2010.

The heat-labile enterotoxin producing enterotoxigenic *E. coli* Labile toxin (ETEC-LT) isolates were fully resistant to amoxicillin and many were resistant to ampicillin, erythromycin and tetracycline, but all were sensitive to gentamicin, nalidixic acid and ofloxacin (Figure 1). The *Shigella* spp. and *Salmonella* spp. isolates were both resistant to ceftriaxone, trimethroprim-sulfamethaxazole, amoxicillin and erythromycin. The *Shigella* spp. isolate was also found to be resistant to tetracycline, whilst the *Salmonella* spp. isolate was also resistant to chloramphenicol. Only four antimicrobials showed no resistance pattern, namely kanamycin, ciprofloxacin, ofloxacin and nalidixic acid.

### Risk factors associated with diarrhoea

In the bivariate analysis, most of the exposure variables examined relating to lack of sanitary practices were associated significantly with diarrhoea. Several factors associated with handwashing were statistically significant, with odds ratios ranging from 4–6. Additional exposure within the home relating to breastfeeding, food preparation and water storage and treatment were statistically significant, with odds ratios ranging from 7–51. Exposure outside the home, such as exposure to other cases of diarrhoea, were also significant, with odds ratios of 5–9 (Table 3).

**TABLE 3:** Bacterial enteric agents isolated in the urban refugee children between the ages of two and five as seen at Eastleigh City Council Health Centre, Nairobi, August–December, 2010.

Bacterial agent	Cases			Controls		
	No. of isolates	%	95% CI	No. of isolates	%	95% CI
***Pathogenic isolates***						
ETEC*	3	7.3	1.5–19.9	1	2.4	0.1–12.9
*Salmonella paratyphi*	2	4.9	0.6–16.5	0	0	0
*Shigella dysenteriae*	1	2.4	0.1–12.9	0	0	0
*Shigella flexneri*	1	2.4	0.1–12.9	0	0	0
***Non-pathogenic isolates***						
*Escherichia coli*	29	70.7	54.5–83.0	37	90.2	76.9–7.3
*Proteus* spp.	2	4.9	0.6–16.5	2	4.9	0.6–6.5
*Providencia* spp.	1	2.4	0.1–12.9	1	2.4	0.1–12.9
*Aeromonas* spp.	2	4.9	0.6–16.5	0	0	0
**Total**	**41**	**-**	**-**	**41**	**-**	**-**

*Source*: Authors’ own construction

* , Enterotoxigenic *Escherichia coli;* CI, Confidence Interval

Eight variables were found to be associated independently with diarrhoeal illness in the final multivariate model. Two were related to the handwashing category (children not washing hands with soap and/or not having a handwashing facility in the home: adjusted odds ratio (aOR) 6); four were related to other exposure within the home (children not exclusively breastfed for their first six months; using a wide-mouthed container to store water; children having eaten food cooked the previous day; and not having a rack for drying dishes in the home: aOR 7–40); and three were related to exposure outside the home (a neighbour having had diarrhoea during the previous two weeks; children sharing a toilet with a diarrhoea patient; and children drinking water from outside the home during the five days before enrolment: aOR 10–40) (Table 4).

**TABLE 4:** Final multivariate model of factors independently associated with diarrhoeal diseases amongst urban refugee children between the ages of two and five seen at Eastleigh City Council Health Centre, Nairobi, August–December, 2010.

Factors	aOR	95 % CI		*p*-Value
		LL	UL	
***Handwashing***				
Child does not wash hands with soap	5.9	2.3	15.6	0.001
No hand washing facility in home	5.5	1.8	18.7	0.005
***Exposure within home***				
Child not exclusively breastfed for first six months	7.6	1.5	39.1	0.020
Use wide-mouthed container to store water	39.8	3.4	470.5	0.003
Child ate food cooked previous day	23.8	3.2	77.1	0.002
No rack for drying dishes in home	6.7	2.3	20.7	0.001
***Exposure outside home***				
Neighbour had diarrhoea two weeks previously	39.9	2.6	623.2	0.008
Child shared toilet with diarrhoea patient	29.9	2.8	322.2	0.005
Child drank water outside home five days before the study	10.0	1.8	57.1	0.009

*Source*: Authors’ own construction

CI, Confidence Interval; LL, Lower Limit; UL, Upper Limit

## Trustworthiness

The findings of this study are based on true findings as spelt out in the research protocol. The observations here outlined are as experienced during the study and are consistent with other research and related studies.

### Reliability and validity of the research

The experimental design of this study is reliable and valid. The procedures used in this research have been tested in other studies as cited in this article. It can be said with the strongest conviction that the results of this study are reproducible in any setting as long as these procedures are employed.

## Discussion

The pathogenic organisms found among these urban refugee children with diarrhoea were ETEC, *Shigella flexneri, Shigella dysenteriae* and *Salmonella paratyphi*. Together, these known pathogens were more common among cases than controls, supporting their aetiological role. These findings agree with a previous study amongst children in Kenya and Senegal,^12,13^ as well as studies done in Bangladesh and Argentina,^14^ which determined that ETEC, *Salmonella* spp. and *Shigella* spp. were responsible for most cases of bacterial diarrhoea in children. *Proteus* spp. and *Providencia* spp., which are generally thought to be non-pathogenic, were isolated from both cases and controls. Of note, however, is that *Aeromonas* spp. were isolated from two diarrhoeal cases and none from the controls. Although the sample size was small, this finding could be significant and can be recommended for further research to determine pathogenicity of this organism, as its presence has also been revealed in other studies.^15^

Approximately 5% of the diarrhoeal cases amongst the urban refugee children were persistent. This finding is consistent with observations from a study conducted in India, which showed that persistent diarrhoea accounted for 5% of cases,^16^ and with a similar study in Zaire.^17^ In Bangladesh, persistent diarrhoea accounted for nearly half of the child diarrhoeal deaths.^18^

This finding agrees with a study done in rural western Kenya.^19^ All pathogenic bacterial isolates were found to be resistant to amoxicillin. ETEC isolates, the most common cause of diarrhoea, were resistant to both amoxicillin and tetracycline, and partially resistant to ampicillin and erythromycin. These antimicrobials are some of the first-line drugs that are prescribed commonly in Kenya; resistance is therefore a cause of concern. This finding is consistent with a related study done in rural western Kenya, which found a high level of isolates to be resistant to the antibiotics prescribed most commonly in Kenya, such as amoxicillin, ampicillin and tetracycline^20^ and was also consistent with a study on antimicrobial resistance conducted in Nigeria.^21^

The mushrooming of clinics and pharmacies in Eastleigh, with no control over the prescription and use of antibiotics, may explain the antibiotic resistance observed. Similarly, previous studies conducted in developing countries have established that, in some locations, antibiotics can be purchased from private hospitals, pharmacies and patent medicine stalls without a prescription, even when the practice is not legal.^22^ Although Oral Rehydration Salt therapy (ORS) is recommended as the primary management for cases of uncomplicated diarrhoea,^23^ only 30% of the diarrhoea cases were managed with ORS, whilst 76% were treated immediately with antibiotics.

We found poor handwashing practices to be a strong risk factor for diarrhoea. This is in agreement with a study done on sanitation which established that washing one’s hands with soap is another important barrier to transmission^24^ and has been cited as being one of the most cost-effective public health interventions.^25^ A number of studies have also shown that handwashing with soap can reduce the incidence of diarrhoeal disease by over 40%.^24^ We also found that most of the children with diarrhoeal illness had eaten food cooked the previous day. Although we were not able to find other studies related to this finding, the observation raises questions on food-storage practices in this community. Use of wide-mouthed containers for water storage was also a strong risk factor for diarrhoea, creating a risk of contamination from the environment. Additionally, non-exclusive breastfeeding was associated significantly with diarrhoeal illness (*p* = 0.015). Despite the fact that our study participants were all above two years old and past the typical age for breastfeeding, this study suggests a long-term benefit of breastfeeding, as has been reported elsewhere.^26^

## Limitations of the study

This study was limited to determining bacterial aetiology with respect to diarrhoea, therefore parasites and viruses which are known to account for most cases of childhood diarrhoea were not investigated. Stool sample processing was not real time since the laboratory is located quite a distance from the study site. However, measures were taken to ensure that sample integrity was maintained, as outlined in the research method and design.

### Recommendations

We identified the need to evaluate the use of antibiotics in diarrhoeal treatment in order to institute measures to control and guide their use and to minimise the risk of antibiotic resistance. There is a need to scale up the use of ORS in the management of diarrhoea, both at home and in the hospital, as well as efforts to improve personal and domestic hygiene. This should include conducting educational campaigns to promote appropriate handwashing using soap, especially at all critical times.

## Conclusions

Infectious diarrhoea remains a major public health problem amongst refugee children under the age of five in Kenya. The pathogenic bacterial agents responsible for diarrhoea amongst these children were ETEC, *Shigella dysenteriae, Shigella flexineri* and *Salmonella paratyphi*. The commonly-prescribed antibiotics prescribed for diarrhoea, such as amoxicillin and ampicillin, are facing threat of resistance from the enteric pathogens.
